# Acute Eosinophilic Pneumonia Secondary to Daptomycin

**DOI:** 10.7759/cureus.19403

**Published:** 2021-11-09

**Authors:** Francisco J Somoza-Cano, Austin Makadia, Massiel P Cruz-Peralta, Lara Zakarna, Eleonora Demyda, Abdul Rahman Al Armashi, Kanchi Patell, Basel Altaqi

**Affiliations:** 1 Internal Medicine, St. Vincent Charity Medical Center, Cleveland, USA; 2 Pulmonary/Critical Care, St. Vincent Charity Medical Center, Cleveland, USA

**Keywords:** daptomycin-induced acute eosinophilic pneumonia, daptomycin induced pneumonitis, septic arthritis, daptomycin, chronic kidney disease (ckd)

## Abstract

Daptomycin is an antimicrobial agent with activity against gram-positive bacteria that is usually reserved for severe infections. Acute eosinophilic pneumonia (AEP) is an increasingly rare side effect that can manifest after its use. Our patient is a 79-year-old male who was admitted for a left total knee arthroplasty infection. After daptomycin was started, he developed AEP. The offending agent was stopped, the appropriate medical treatment was given, and his symptoms significantly improved. This case illustrates an uncommon side effect of daptomycin. Prompt medical recognition is paramount for medication discontinuation and adequate medical care.

## Introduction

Daptomycin is a lipopeptide antibiotic that acts by inserting the lipophilic tail of its chemical structure into the bacterial cell wall. This depolarizes the bacterial cell membrane, increases potassium efflux, and finally produces bacterial cell death [1]. The most common adverse effects include bleeding, blistering, burning, coldness, discoloration of the skin, hives, and itching [2]. Acute eosinophilic pneumonia (AEP) was added to its potential adverse effects after the Food and Drug Administration (FDA) Adverse Event Reporting System database revealed seven definite, 13 probable, and 38 possible cases of eosinophilic pneumonia caused by daptomycin [2,3]. We present a case where a patient developed AEP after daptomycin therapy.

## Case presentation

A 79-year-old male with a past medical history of chronic infection of a left knee prosthesis, hypertension, and chronic kidney disease stage 3A presented to the wound care clinic after two days of subjective fever that partially improved with acetaminophen. He denied any associated symptoms. The patient had recently undergone multiple left knee revisions and received several antibiotics in an attempt to treat the draining chronic left knee infection (Table 1).

**Table 1:** Previous antibiotic regimens in alphabetical order Our patient had been on several antibiotic regimens for his chronic knee infection. Medications were either stopped due to a completed regimen, failed to improve the clinical status, or adverse reactions. BID: twice a day; TID: three times a day; PO: by mouth; IV: intravenously.

Medication	Dosage	Frequency, Route	Start date	Stop date
Amoxicillin/clavulanic acid	875 mg	BID, PO	3/20/18	3/21/18
Amoxicillin/clavulanic acid	875 mg	BID, PO	6/21/18	6/22/18
Cefazolin	1 g	Q8H, IV	8/1/18	three doses
Amoxicillin/clavulanic acid	500 mg	BID, PO	11/18/20	11/21/20
Cefazolin	1 g	BID, IV	3/16/21	3/17/20
Cefazolin	1 g	TID, IV	3/16/17	5/16/17
Cefazolin	1 g	Once, IV	11/20/20	
Cefepime	2 g	BID, IV	5/17/17	19/17/17
Ceftriaxone	2 g	Daily, IV	3/17/21	3/22/21
Ceftriaxone	2 g	Daily, IV	11/20/20	11/25/20
Ceftriaxone	2 g	Daily, IV	7/2/20	7/6/20
Ciprofloxacin	400 mg	BID, IV	5/10/21	5/14/21
Daptomycin	675 mg	Daily, IV	6/9/21	6/22/21
Ertapenem	500 mg	Daily, IV	6/9/2021	6/22/2021
Vancomycin	1.5 g	Daily, IV	11/20/20	11/25/20

He developed severe allergic reactions (urticarial rashes and angioedema) to cephalexin and ciprofloxacin trimethoprim/sulfamethoxazole. Subsequently, 11 days prior to presentation, he was started on ertapenem and daptomycin. Moreover, before intravenous daptomycin and ertapenem were started, he had not received additional antibiotics for over a month. The patient had no previous history of pulmonary diseases and he denied any exposure to pulmonary irritants. Upon admission, the patient had a Hickman catheter for long-term antibiotic use without signs of acute inflammation. He had wheezing throughout the bilateral lung fields but no crackles. A draining tract with serosanguinous fluid drainage was observed on his left knee. His physical exam was otherwise unremarkable. The initial laboratory work demonstrated moderate anemia, normal white blood cells but with bandemia, and an elevated erythrocyte sedimentation rate and C-reactive protein (Table 2).

**Table 2:** Initial work-up WBC: white blood cells; RBC: red blood cells; MCV: mean corpuscular volume; MCH: mean corpuscular hemoglobin; MCHC: mean corpuscular hemoglobin concentration; BUN: blood urea nitrogen; AST: aspartate aminotransferase; ALT: alanine aminotransferase; PT: prothrombin time; PTT: Partial thromboplastin time; INR: international normalized ratio; ESR: erythrocyte sedimentation rate; CRP: C-reactive protein.

Lab Finding	Result	Reference range	Units
Complete Blood Count			
WBC	10.9	3.9 - 11	x 10^3
Lymphocytes	6	20 - 50	%
Neutrophils	70	40 - 80	%
Bands	10	0 - 5	%
Monocytes	8	2 - 12	%
Eosinophils	2	0 - 5	%
Basophils	1	0 - 1	%
RBC	3.06	3.5 - 5.5	x 10^6
Hemoglobin	9.2	14 - 16.5	g/dL
Hematocrit	26.9	39 - 55	%
MCV	87	80 - 100	fL
MCH	29.4	25.4 - 34.6	Pg
MCHC	33.5	31.5 - 36.5	g/dL
Platelets	204	140 - 440	x 10^3
Complete Metabolic Panel			
Sodium	141	136 - 145	mg/dL
Potassium	3.88	3.5 - 5.1	mg/dL
Chloride	110	98 - 107	mg/dL
Calcium	8.51	8.5 - 10.1	mg/dL
Magnesium	1.9	1.6 - 2.6	mg/dL
Creatinine	1.3	0.7 - 1.3	mg/dL
BUN	29.9	7 - 18	mg/dL
AST	150	15 - 37	U/L
ALT	73	13 - 61	U/L
Albumin	4	3.4 - 5.0	g/dL
PT	16	9.0 - 12.5	seconds
PTT	29.6	23.9 - 31.8	seconds
INR	1.2	0.00 - 1.2	IU
Lactic Acid	3.4	0.4 - 2.0	mmol/L
Inflammatory Markers			
ESR	102	0 - 20	mm/h
CRP	144	0.0 - 3	mg/L
Procalcitonin	0.1	<0.05	ng/mL

**Figure 1:**
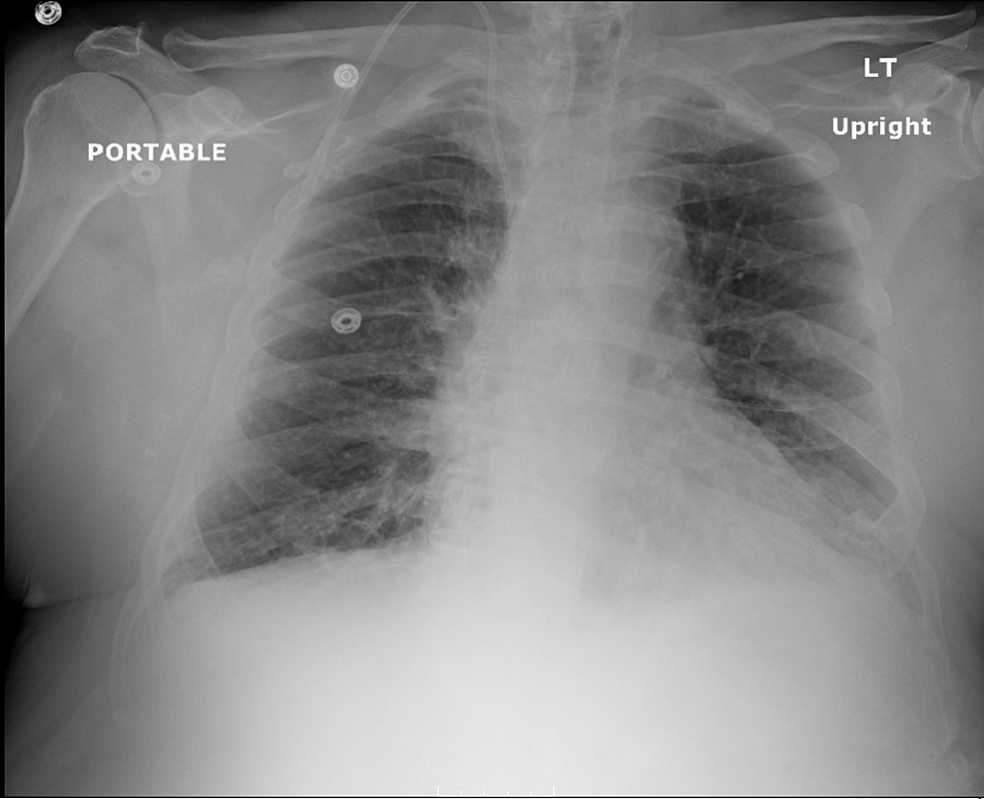
Initial chest x-ray showing new diffuse interstitial opacities

A chest x-ray showed new diffuse interstitial opacities (Figure 1). A single anteroposterior portable chest X-ray was obtained on admission. Compared to a chest X-ray nine months prior, there were new diffuse branching interstitial opacities extending outward from hila associated with additional circular interstitial opacities. These were likely representing peribronchovascular interstitial thickening. There may be a trace of left pleural effusion without right pleural effusion. Additionally, the aorta was atherosclerotic and a right internal jugular central venous catheter terminating at the cavoatrial junction was seen in situ.

**Figure 2:**
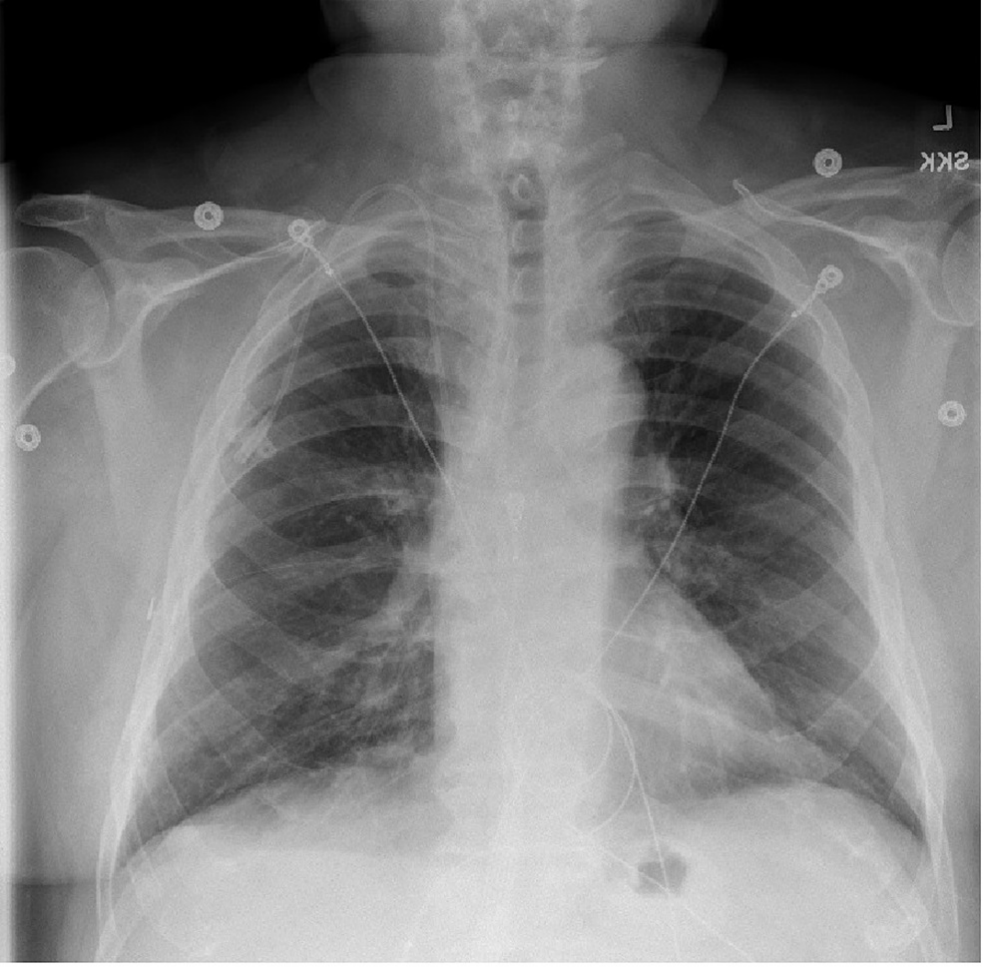
Follow-up chest x-ray showing resolution of the interstitial markings

On the second day of admission, the patient started having worsening shortness of breath, desaturation, tachycardia, and persistent fever. Examination of the lungs revealed bilateral crackles while the lower limbs and the trunk showed a new reticulopapular, non-pruritic rash. Repeat complete blood count (CBC) revealed new-onset eosinophilia. A computed tomography (CT) scan of the chest showed diffuse reticulonodular opacities in the lungs with peripheral predominance, areas of ground-glass opacities, and nodularities. Daptomycin AEP was suspected and the antibiotic was promptly discontinued and meropenem was started. Methylprednisolone and inhaled breathing treatments were ordered and the patient’s symptoms significantly improved thereafter. A repeat chest x-ray one month later showed resolution of the interstitial markings (Figure 2). One month after the initial presentation, a repeat chest x-ray was obtained. The lungs appeared clear without apparent pleural effusion. No active disease in the chest was identified.

## Discussion

Daptomycin is a lipopeptide antibiotic derived from the fermentation of *Streptomyces roseosporus* [4]. After its release to the market in 2003, occasional case reports emerged documenting AEP after its use. A post-marketing analysis confirmed its potential to cause AEP [2]. Nonetheless, this side effect remains an uncommon occurrence. To date, few cases in the United States have been described [3].

The exact pathophysiological mechanism behind AEP induced by daptomycin is yet to be elucidated. However, the most accepted theory relies on daptomycin’s ability to bind to the pulmonary surfactant, accumulate within the alveoli, and produce epithelial injury and inflammation. Consequently, there is the recruitment of the T-helper 2 lymphocytes by the alveolar macrophages. This in turn releases interleukin-5, increasing eosinophil recruitment and production. Then, alveolar macrophages release a potent eosinophil chemoattractant, eotaxin, leading to further aggregation of these cells within the lung [3-5].

AEP after daptomycin therapy has been classified into definite, probable, possible, and unlikely [3,6]. The diagnosis criteria include concurrent exposure to daptomycin, fever, dyspnea with increased oxygen requirements, new infiltrates on x-ray or chest CT scan, clinical improvement following daptomycin withdrawal and bronchoalveolar lavage (BAL) with >25% eosinophils make the diagnosis criteria [6]. Our patient met all of the aforementioned criteria except the eosinophils in the BAL, which was not done due to an almost prompt response to steroid therapy and medication discontinuation. In the original FDA’s analysis, only seven of the 62 cases met all criteria [6]. The remaining cases were deemed as probable or possible due to the causal relationship between daptomycin and AEP and were included in the analysis nonetheless [3,6-10].

Moreover, using the Naranjo scale for medication adverse effects, our patient had a score of 6 points, making the diagnosis probable [11]. Additionally, Bradford-Hill's criteria for a causal relationship applied to our case support the connection between daptomycin and AEP [12]. Proven evidence of daptomycin-induced AEP, a temporal relationship between initiation of therapy and symptom onset, worsening course with medication continuation, and evidence of reversibility in a variety of different patient populations support the aforementioned causality [12].

The initial management for AEP secondary to daptomycin includes medication discontinuation and supportive measures [3,13-15]. Empiric antibiotics and systemic glucocorticoid therapy have also proved useful. Clinical trials have not tackled these interventions but worsening dyspnea and respiratory failure have been documented without steroid therapy. Even if steroids are not promptly given, the patients usually improve within 48 hours even after delayed corticosteroid therapy [3,6-10,13-15]. In our case, the acute onset peripheral eosinophilia, the prompt response to corticosteroids, and medication discontinuation, support the diagnosis of AEP secondary to daptomycin.

## Conclusions

AEP remains a rare and underknown adverse reaction after daptomycin therapy. Elderly patients with chronic kidney disease and receiving high doses are, particularly at risk. Our case illustrates the importance of a high index of suspicion for early recognition of this side effect, especially in at-risk patient populations. It is imperative for clinicians to be familiarized with the condition in order to rapidly recognize its presentation and treat it appropriately.
